# Experimental Tuberculosis, Human and Bovine, in the Domestic Animals1Presented at the thirty-seventh annual meeting of the American Veterinary Medical Association, held at Detroit, Mich., September 4, 1900.

**Published:** 1900-12

**Authors:** R. R. Dinwiddie

**Affiliations:** Arkansas Agricultural Experiment Station


### THE JOURNAL

OF

### COMPARATIVE MEDICINE AND VETERINARY ARCHIVES.

VOL. XXI. *DECEMBER, 1900.* No. 12.

### EXPERIMENTAL TUBERCULOSIS, HUMAN AND BOVINE, IN THE DOMESTIC ANIMALS.[1]

BY R. R. DINWIDDIE, M.D., V.S.,
ARKANSAS AGRICULTURAL EXPERIMENT STATION.

PRACTICALLY all the domestic animals are susceptible to inoculation tuberculosis—horses, cattle, sheep, goats, pigs, cats, dogs, and poultry—although with a varying degree of susceptibility. The susceptibility, indeed, varies according to the origin of the tubercular material used for the inoculation, but does not, contrary to what we might expect, exactly correspond to the liability to the spontaneous or naturally acquired disease. In the guinea-pig we have, perhaps, the most striking example of this anomaly. Although the most susceptible of all animals to inoculation with mammalian tubercle, it seems to be practically exempt from spontaneous disease of this kind, even when kept under the most unfavorable conditions of environment. On the other hand, as we shall see from some experiments of my own which I intend to refer to later, cattle, although, as is well known, the most prone of all the domestic animals to spontaneous tuberculosis, do not show any greater susceptibility to the inoculation disease than either sheep or pigs. When the less virulent variety of tubercle bacilli which we obtain from the human consumptive is employed they show a susceptibility even less in degree than these latter animals. I propose to bring forward experimental proof of this fact first before attempting to furnish the explanation.

Cases of tuberculosis naturally acquired have been recorded in pigs, dogs, cats, horses, sheep, and goats in the order of frequency somewhat as named. This includes about all the species which

[1] Presented at the thirty-seventh annual meeting of the American Veterinary Medical Association, held at Detroit, Mich., September 4, 1900.

come under the observation of the veterinarian. The disease in these animals which I have just named presents two features which I wish especially to refer to. The first is its relative infrequency. In pigs, outbreaks, if they may be so called, are observed in which many animals on a farm may be found diseased. In the other species of animals just mentioned only isolated cases occur, and these are of extreme rarity.

The second feature I allude to is this, that in these animals the contagion seems generally to have an origin extraneous to the species concerned. Pigs become infected by feeding upon milk from tuberculous cows; cats and dogs in the same way, or more frequently by consuming the sputa of phthisical human beings. A few cases of tuberculosis in horses are on record in which a probability of infection from association with tuberculous cows is referred to, while in other cases no history is obtainable. In sheep and goats the source of infection is also uncertain in the very few cases of spontaneous tuberculosis which have been described in these animals.

While it is impossible to deny that, from close cohabitation, a transmission of the disease from animal to animal of the same species may occur, we know from the facts just stated that such transmission must be extremely rare. Whether or not part of the tuberculosis found in pigs may have its source in other animals of the same kind previously infected by tuberculous cows' milk must remain in question. On this point we have really no information. We can hardly assert, therefore, that tuberculosis prevails as a plague spread by cohabitation and perpetuated by transmission in the same species except in the case of mankind, cattle, and poultry.

I have brought forward these points in order to show that if we hypothetically assume the existence of races of tubercle bacilli corresponding to the zoological species in which they are chiefly parasitic, we can find grounds for such an assumption among the mammalia only in the human race and the bovine, the conditions on which we believe such an evolution to depend—long and exclusive parasitism in one host-species—being lacking in the other species of domestic animals. Such a theory of race differentiation finds some support from facts which have been observed in connection with other species of pathogenic bacteria, among which I may especially mention the bacilli of hemorrhagic septicæmia, which includes a group of organisms presenting close similarity in morphological and biological characters, but differing in the zoological species toward which their pathogenic activity is chiefly directed. In tuberculosis of fowls—avian tuberculosis—the existence of a variety of tubercle bacilli differing from the mammalian is well recognized from cultural and morphological peculiarities as well as by experimental inoculations in animals.

Some slight differences, morphological and cultural, have been observed between cultures of tubercle bacilli obtained from sputum and those isolated from bovine tubercular products. A more striking difference is found when their virulence is tested by inoculation of certain animals. The main facts which we possess in this connection have been brought to light only recently, and by the investigations of Theobald Smith, who has made an extensive series of inoculations with sputum and bovine tubercle-bacilli cultures on cattle and on rabbits. By these comparative tests with cultures the greater virulence of those of bovine origin for these animals has been clearly shown. Comparative tests with cultures had not previously been made, but a study of the records of the earlier inoculations made with disease products shows that this higher virulence of bovine tubercle toward rabbits and cattle had been recognized even before Koch's discovery of the specific bacillus.

In a previous report[1] I have endeavored to gather together from the scattered records of inoculation experiments, both early and recent, the data upon which we might base an opinion as to the relative virulence for the domestic animals of human and bovine tubercle or artificial cultures of the bacilli from these respective sources. I have also in that report given the details of some experiments of my own in which cattle, pigs, sheep, and poultry were inoculated with crude tubercular material or with artificial cultures. The conclusions arrived at from a study of all the data obtainable may be best expressed by the following brief quotation: "To sum up the matter in a few words, it may be said that bovine tubercle has been shown to be more virulent than the human variety for cattle, sheep, goats, and rabbits, while no distinction has been shown in the case of horses, pigs, cats, and dogs." It should be stated that the data upon which these conclusions were based, though embracing all furnished to us by the literature of tuberculosis experimentation, was far from convincing, the evidence in the case of cattle alone, among the domestic animals, being at all complete, although even here to some extent conflicting.

[1] Bulletin No. 57, Arkansas Agricultural Experiment Station.

Referring to my own experiments, already published, a repetition of which, however, I desire to avoid here as far as possible, I may make the following statement, expressing the results obtained in the briefest manner possible: Cattle showed a greater susceptibility to inoculation with bovine tubercular products than to tubercular sputa of man. This difference was more strongly brought out by inoculation of artificial cultures, on account, presumably, of the larger doses employed. The results of inoculation from sputa were local only, and in no case exhibited the characters of a progressive disease manifestly interfering with the health of the animal. Inoculations from diseased cattle occasioned more extensive lesions, although in several animals apparently also of a non-progressive character. In other cases the disease was either acute and rapidly fatal, or approached the characters of the naturally acquired disease.

Sheep showed a high susceptibility to inoculation with a bovine culture, while their susceptibility to sputum cultures remained in question.

Pigs were found to be readily infected and prone to suffer from a generalized disease by inoculation and feeding with both varieties of tubercle or tubercle bacilli in cultures. A difference in susceptibility to the two varieties was not demonstrable.

Fowls—ordinary farm-yard chickens of a few months old in my experiments—showed themselves entirely invulnerable to massive intraperitoneal injections of cultures from both sources.

I have since then supplemented these experiments with others, using in this instance artificial cultures only. The use of artificial cultures possesses the advantage that the doses of inoculating material can be better compared and much larger amounts injected than is possible with the natural disease products. In the tubercular disease of cattle the specific bacilli are frequently so rare that their demonstration is difficult, and the numbers present in any material which can be obtained suitable for injection apparently insufficient to produce a decided pathological effect on the larger animals. I have, accordingly, in my later experiments made use of artificial cultures injected in rather massive doses into the peritoneal cavity. Contrary to what might be supposed, this route of infection, in cattle at least, seems to give rise to more extensive and progressive disease than the pulmonary.

In order to make the comparison more thorough, cultures of the bacilli of human pulmonary phthisis from separate families and cultures from diseased cattle of different herds were employed, ten separate cultures in all. Seven of these cultures were isolated by myself by passage through the guinea-pig in the usual way—four sputum cultures and three bovine—while three others—bovine cultures—I obtained in the laboratory of comparative pathology, Harvard Medical School, through the courtesy of Professor Smith. They have been tested on calves, pigs, sheep, and cats, and incidentally also on guinea-pigs. In this paper I propose to give the details of the completed experiment on pigs, and the results of the inoculations with sputum cultures on calves and sheep, the experiments with bovine cultures on these latter animals being as yet incomplete.

EXPERIMENT I. *Tuberculosis Complicated with Swine-pest and Swine-plague.* Although this experiment from the point of view from which it was undertaken must be considered a failure, it possesses sufficient interest of its own to justify its repetition here, showing, as it does, the influence of pre-existing pulmonary lesions on the course of tubercular disease subsequently acquired.

Three pigs, each about six months old, numbered in records 10, 11, and 12, were inoculated with sputum cultures, while another of the same size was inoculated with a bovine culture. Two of these pigs came from a farm on which hog-cholera was prevailing, while the other two, after undergoing some immunizing experiments, had been fed in my own pens with cultures of the bacillus of this disease. None of them showed at the time any distinct symptoms of being infected. Of the cultures employed, Sputum I., inoculated to Pig 10, was the first transfer from the guinea-pig, a six weeks' old culture; II. and III. were second transfers, twenty-four days old, and also recently isolated. All were large growths on 5 per cent. glycerin-serum. Culture, Bovine I., used on Pig 9, was the eighth transfer, a smaller growth of one month's duration on the same medium. It had been isolated one year previously, and grown since that time on serum. About a pin-head-sized piece of each of the cultures rubbed up in sterile normal salt solution was injected in each case into the peritoneal cavity in the hypogastric region on May 12, 1899. The two lots were kept in separate pens.

All these pigs soon began to pine. Pig 12 suffered from intense dyspnœa with paroxysms of coughing for two or three weeks before it died, July 21st—that is, two months and one week after inoculation. Pigs 11 and 9 died July 30th and August 15th respectively, while Pig 10 was killed for examination September 25th, four and a half months after inoculation.

I cannot give here in detail the post-mortem findings in each case, nor is this necessary, since the abdominal lesions were nearly alike in all. These represent the local lesions, since the inoculated fluid becomes distributed over the whole serosa. They occur as small yellow, seed-like nodules, distributed more or less thickly in the serous covering of the abdominal cavity and contained organs. The distribution is unequal, the great omentum and sulci between the coils of the large intestine containing the largest number and also those of largest size. This, I may state here, applies to all animals which I have tested by this method of inoculation. These nodules occur in varying number on the peritoneal surface of the diaphragm, stomach, and spleen. They are rarer on the walls of the intestine. Some are apparently within the membrane, others at the free end of delicate connective tissue fringes. The surface of the liver usually shows, in addition, a number of yellow non-projecting spots of the size of a pin-head or larger, which are also tubercular changes.

Of the four pigs of this experiment one only—that which lived the longest—showed any involvement of the abdominal lymph nodes, those situated between the coils of the large intestine. In the lungs the most interesting changes were found. In two cases at least—11 and 12—the condition was plainly one of swine-plague pneumonia with pulmonary tuberculosis superadded. In No. 12 both lungs were solidified, and on section showed many pus-containing cavities from the size of a walnut down. Other areas were gray on section, hepatized, but free from cavities, while in other places, again, where the disease was less advanced, the lung, though hepatized, was less resistant to the knife, dark red in color, and dotted with small necrotic foci, a picture of a common type of pneumonia seen in contagious swine disease associated with the swine-plague bacillus of Smith. In Pig 11 the greater part of both lungs were also involved ; the section was white and caseous looking, purulent fluid oozed from the smaller bronchi, a few nut-sized cavities were present, and many points of softening, indicating the beginning of abscess formation. There was also some pleurisy with adhesions. Distinct tubercles were not found in the lungs of either of these animals, but the proof of the tubercular complication was furnished by the microscopical examination. Cover-glass preparations from all parts of the incised lung showed tubercle bacilli in numbers comparable to what we find in the sputa of advanced pulmonary phthisis of man. Rabbits inoculated from the lungs of each of the pigs died of swine-plague bacilli septicæmia. In the large intestine of both, the ulcerous lesions which we regard as characteristic of swine-pest or "hog-cholera" were present. Moreover, from other animals of the same herd the bacillus of this latter disease was isolated.

Pig 10, also inoculated with a sputum culture, and killed in four and a half months, was found free from extraneous disease. The tubercular changes in the thoracic cavity were limited to the supra-sternal lymph nodes.

Pig 9, inoculated with the bovine culture, showed a more extensive involvement of the abdominal serosa than the other three, tubercular pus-pockets in the peribronchial lymph nodes and complete invasion of the lungs with small yellow tubercles not yet broken down in ulceration. In the cæcum were a number of superficial swine-pest ulcers.

Having digressed somewhat to give this experiment in detail, it is unnecessary to give more than the briefest comment upon it. I should notice in particular the extraordinary rapidity of the extension of the tubercular infection from the peritoneum to the already diseased lung, the absence of distinct tubercles in the lung, and the enormous multiplication of tubercle bacilli in the diseased pulmonary tissue or pathological secretions of the bronchioles. As a comparative test it may be noticed that the pig receiving the bovine culture showed more extensive tubercular disease than the one inoculated with the sputum culture and uncomplicated with swine-plague infection.

EXPERIMENT II. *Comparative Test of Sputum and Bovine Cultures on Pigs. Inoculation with Sputum Cultures.* As the cultures used in this experiment were the same as those used for other inoculations yet to be described, their history may be given here once for all.

Sputum I. Fourth transfer, one month old, five months from animal.

Sputum II. Fifth transfer, three to four weeks old, five months from animal.

Sputum III. Fifth transfer, four weeks old, six months from animal.

Sputum IV. Second transfer, four weeks old, three and a half months from animal.

Of the patients from whom the sputum was obtained it need only be said that all were ordinary cases of pulmonary disease in more or less advanced stage, three of which have since died, while the fourth has been lost sight of.

All cultures were made on 5 per cent. glycerin serum (beef), and showed a vigorous growth when used. They were all, therefore, recently isolated cultures and presumably possessed of their full original virulence.

Four healthy four-months-old pigs received each an inoculation similar to that already described. The inoculations were made on August 12, 1899, and the animals kept under observation for nearly a year, when they were killed for examination, in July, 1900. They were kept constantly penned, and three of them ultimately became unthrifty and much emaciated, partly due, perhaps, to the lack of sufficient green food.

The dissection of all of these animals revealed changes such as spoken of before in the abdominal cavity, but the nodules were smaller in size, not very numerous, and all calcareous. In one the lungs contained numerous small yellow tubercles, and, in the apices, areas of consolidation with formation of cavities, recalling the condition found in the same disease in man. In two others the tubercles in the lung were less numerous and showed merely as small calcareous masses the size of millet seed enclosed in cysts, from which they were easily shelled out and without marked hyperplasia around them. They were good examples of what may be called healed tubercles. In the fourth animal no pulmonary changes were found. In these old calcareous tubercles the specific bacilli could not be demonstrated;[1] in the apical abscess cavity they were numerous.

(To be continued.)

[1] They also proved non-infective when inoculated to the guinea-pig.

